# 2-Methyl­amino-5-nitro­benzoic acid

**DOI:** 10.1107/S160053681001946X

**Published:** 2010-05-29

**Authors:** Abdul Rauf Raza, Syeda Laila Rubab, M. Nawaz Tahir

**Affiliations:** a Department of Chemistry, University of Sargodha, Sargodha, Pakistan; b Department of Physics, University of Sargodha, Sargodha, Pakistan

## Abstract

The title compound, C_8_H_8_N_2_O_4_, is almost planar (r.m.s. deviation = 0.037 Å) and an intra­molecular N—H⋯O hydrogen bond generates an *S*(6) ring. In the crystal, inversion dimers linked by pairs of O—H⋯O hydrogen bonds generate *R*_2_^2^(8) loops. Inter­molecular N—H⋯O hydrogen bonds (involving the same H atom that forms the intra­molecular hydrogen bond) link the dimers into infinite sheets lying parallel to (102).

## Related literature

For background to the medicinal properties of benzodiazepines, see: Blank *et al.* (2009 ▶); Kamal *et al.* (2010 ▶). For a related structure, see: Dhaneshwar & Pant (1972 ▶). For graph-set theory, see: Bernstein *et al.* (1995 ▶).
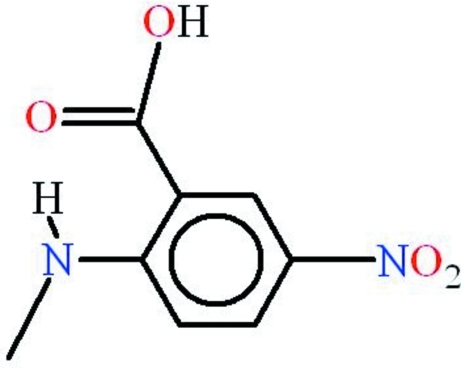


## Experimental

### 

#### Crystal data

C_8_H_8_N_2_O_4_
*M* *_r_* = 196.16
Monoclinic, 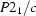

*a* = 7.2541 (12) Å
*b* = 14.037 (2) Å
*c* = 8.5972 (14) Å
β = 103.673 (6)°
*V* = 850.6 (2) Å^3^
*Z* = 4
Mo *K*α radiation
μ = 0.13 mm^−1^
*T* = 296 K
0.34 × 0.12 × 0.10 mm

#### Data collection

Bruker Kappa APEXII CCD diffractometer
Absorption correction: multi-scan (*SADABS*; Bruker, 2005 ▶) *T*_min_ = 0.979, *T*_max_ = 0.988
6739 measured reflections
1667 independent reflections
931 reflections with *I* > 2σ(*I*)
*R*_int_ = 0.051

#### Refinement

*R*[*F* ^2^ > 2σ(*F* ^2^)] = 0.051
*wR*(*F* ^2^) = 0.156
*S* = 0.95
1667 reflections
129 parameters
H-atom parameters constrained
Δρ_max_ = 0.25 e Å^−3^
Δρ_min_ = −0.27 e Å^−3^

### 

Data collection: *APEX2* (Bruker, 2007 ▶); cell refinement: *SAINT* (Bruker, 2007 ▶); data reduction: *SAINT*; program(s) used to solve structure: *SHELXS97* (Sheldrick, 2008 ▶); program(s) used to refine structure: *SHELXL97* (Sheldrick, 2008 ▶); molecular graphics: *ORTEP-3 for Windows* (Farrugia, 1997 ▶) and *PLATON* (Spek, 2009 ▶); software used to prepare material for publication: *WinGX* (Farrugia, 1999 ▶) and *PLATON*.

## Supplementary Material

Crystal structure: contains datablocks global, I. DOI: 10.1107/S160053681001946X/hb5463sup1.cif

Structure factors: contains datablocks I. DOI: 10.1107/S160053681001946X/hb5463Isup2.hkl

Additional supplementary materials:  crystallographic information; 3D view; checkCIF report

## Figures and Tables

**Table 1:** Hydrogen-bond geometry (Å, °)

*D*—H⋯*A*	*D*—H	H⋯*A*	*D*⋯*A*	*D*—H⋯*A*
N1—H1⋯O1	0.86	2.03	2.694 (3)	134
N1—H1⋯O4^i^	0.86	2.52	3.165 (3)	133
O2—H2⋯O1^ii^	0.82	1.86	2.679 (3)	177

Symmetry codes: (i) 
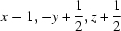
; (ii) 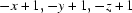.

**Fig. 1.**
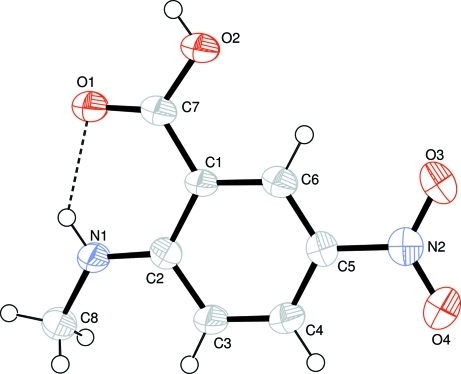
View of (I) with displacement ellipsoids drawn at the 50% probability level. The dotted line indicate the intramolecular H-bond.

**Fig. 2.**
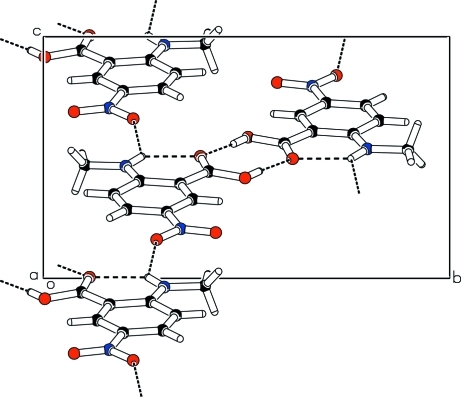
The partial packing diagram of (I), which shows that molecules form polymeric chains extending along the b-axis.

## supplementary crystallographic information

### Comment

The benzodiazepines constitute a very diverse class of heterocyclic compounds with plethora of biological activities such as anti-caner (Kamal *et al.*, 2010) and anti-HIV (Blank *et al.*, 2009) agent. The title compound (I, Fig. 1) was synthesized as a precursor for the synthesis of benzodiazepine derivative and it will also be utilized for the metal complexation.

The crystal structures of *N*-methylanthranilic acid (II) (Dhaneshwar & Pant, 1972) has been published. The title compound differs from (II) due to substitution of nitro group at at position five.

The asymmetric unit of title compound is essentially planar with r. m. s. deviation of 0.0366 Å from the least square plane of (C1—C8/N1/N2/O1/O2/O3). There exist a S(6) ring motif (Bernstein *et al.*, 1995) due to N—H···O type of intramolecular H-bondings. The molecules are dimerised due to inversion related O—H···O type of H-bondings with R_2_^2^(8) ring motifs. The dimers are interlinked in the form of infinite two dimensional polymeric sheets due to H-bonding of N—H···O type (Fig. 2).

### Experimental

To HNO_3_ (1.83 g, 0.03 mol) taken in an ice chilled round bottom flask the H_2_SO_4_ (2.6 g, 0.026 mol) was added as drops with constant stirring. A solution of N-methylanthranilic acid (2 g, 0.01 mol) in EtOAc (25 ml) was added as drops to the nitrating mixture in ice chilled water bath and stirred for half an hour followed by 3 hours reflux. The reaction mixture was neutralized and extracted with EtOAc (3 × 30 ml). The organic layer was dried over anhydrous Na_2_SO_4_ and concentrated under reduce pressure that afforded purple needles of (I) upon standing.

### Refinement

Although H atoms were appeared in difference Fourier map but were positioned geometrically with (C-H = 0.93–0.96 and O-H = 0.82 Å) and refined as riding with *U*_iso_(H) = x*U*_eq_(C), where x = 1.5 for methyl and hydroxy H-atoms and x = 1.2 for other H atoms.

### Crystal data

**Table:**

C_8_H_8_N_2_O_4_	*F*(000) = 408
*M**_r_* = 196.16	*D*_x_ = 1.532 Mg m^−^^3^
Monoclinic, *P*2_1_/*c*	Mo *K*α radiation, λ = 0.71073 Å
Hall symbol: -P 2ybc	Cell parameters from 931 reflections
*a* = 7.2541 (12) Å	θ = 2.8–26.0°
*b* = 14.037 (2) Å	µ = 0.13 mm^−^^1^
*c* = 8.5972 (14) Å	*T* = 296 K
β = 103.673 (6)°	Needle, colorless
*V* = 850.6 (2) Å^3^	0.34 × 0.12 × 0.10 mm
*Z* = 4	

### Data collection

**Table:**

Bruker Kappa APEXII CCD diffractometer	1667 independent reflections
Radiation source: fine-focus sealed tube	931 reflections with *I* > 2σ(*I*)
graphite	*R*_int_ = 0.051
Detector resolution: 7.50 pixels mm^-1^	θ_max_ = 26.0°, θ_min_ = 2.8°
ω scans	*h* = −8→8
Absorption correction: multi-scan (*SADABS*; Bruker, 2005)	*k* = −17→12
*T*_min_ = 0.979, *T*_max_ = 0.988	*l* = −10→10
6739 measured reflections	

### Refinement

**Table:**

Refinement on *F*^2^	Primary atom site location: structure-invariant direct methods
Least-squares matrix: full	Secondary atom site location: difference Fourier map
*R*[*F*^2^ > 2σ(*F*^2^)] = 0.051	Hydrogen site location: inferred from neighbouring sites
*wR*(*F*^2^) = 0.156	H-atom parameters constrained
*S* = 0.95	*w* = 1/[σ^2^(*F*_o_^2^) + (0.0901*P*)^2^] where *P* = (*F*_o_^2^ + 2*F*_c_^2^)/3
1667 reflections	(Δ/σ)_max_ < 0.001
129 parameters	Δρ_max_ = 0.25 e Å^−^^3^
0 restraints	Δρ_min_ = −0.27 e Å^−^^3^

### Special details

**Table:**

Geometry. Bond distances, angles etc. have been calculated using the rounded fractional coordinates. All su's are estimated from the variances of the (full) variance-covariance matrix. The cell esds are taken into account in the estimation of distances, angles and torsion angles
Refinement. Refinement of *F*^2^ against ALL reflections. The weighted *R*-factor *wR* and goodness of fit *S* are based on *F*^2^, conventional *R*-factors *R* are based on *F*, with *F* set to zero for negative *F*^2^. The threshold expression of *F*^2^ > σ(*F*^2^) is used only for calculating *R*-factors(gt) *etc*. and is not relevant to the choice of reflections for refinement. *R*-factors based on *F*^2^ are statistically about twice as large as those based on *F*, and *R*- factors based on ALL data will be even larger.

### Fractional atomic coordinates and isotropic or equivalent isotropic displacement parameters (Å^2^)

**Table:**

	*x*	*y*	*z*	*U*_iso_*/*U*_eq_	
O1	0.5419 (3)	0.38658 (12)	0.5049 (2)	0.0518 (7)	
O2	0.7127 (3)	0.49628 (11)	0.4158 (3)	0.0573 (8)	
O3	1.2269 (3)	0.42409 (14)	0.1896 (3)	0.0729 (9)	
O4	1.3096 (3)	0.27893 (14)	0.1606 (3)	0.0672 (8)	
N1	0.6259 (3)	0.20268 (13)	0.4642 (3)	0.0468 (8)	
N2	1.2080 (3)	0.33798 (16)	0.2050 (3)	0.0520 (9)	
C1	0.7967 (3)	0.33700 (15)	0.3914 (3)	0.0356 (8)	
C2	0.7635 (3)	0.23711 (16)	0.4028 (3)	0.0380 (8)	
C3	0.8868 (3)	0.17368 (17)	0.3470 (3)	0.0421 (9)	
C4	1.0304 (4)	0.20647 (17)	0.2851 (3)	0.0445 (9)	
C5	1.0588 (3)	0.30381 (17)	0.2745 (3)	0.0412 (9)	
C6	0.9435 (4)	0.36766 (16)	0.3270 (3)	0.0405 (8)	
C7	0.6728 (4)	0.40694 (16)	0.4424 (3)	0.0404 (9)	
C8	0.5800 (4)	0.10286 (17)	0.4683 (4)	0.0530 (11)	
H1	0.55963	0.24247	0.50427	0.0562*	
H2	0.63612	0.53160	0.44325	0.0859*	
H3	0.86864	0.10833	0.35312	0.0505*	
H4	1.10970	0.16375	0.24988	0.0534*	
H6	0.96473	0.43263	0.31904	0.0486*	
H8A	0.68822	0.06878	0.52857	0.0796*	
H8B	0.54575	0.07839	0.36101	0.0796*	
H8C	0.47552	0.09499	0.51789	0.0796*	

### Atomic displacement parameters (Å^2^)

**Table:**

	*U*^11^	*U*^22^	*U*^33^	*U*^12^	*U*^13^	*U*^23^
O1	0.0570 (13)	0.0296 (10)	0.0792 (14)	0.0016 (8)	0.0367 (11)	0.0003 (9)
O2	0.0657 (13)	0.0256 (11)	0.0921 (16)	0.0005 (9)	0.0418 (12)	−0.0015 (9)
O3	0.0793 (16)	0.0436 (12)	0.1119 (19)	−0.0089 (10)	0.0550 (14)	0.0108 (12)
O4	0.0582 (13)	0.0610 (13)	0.0958 (17)	0.0082 (10)	0.0448 (12)	0.0052 (12)
N1	0.0541 (14)	0.0254 (11)	0.0716 (16)	−0.0034 (10)	0.0363 (13)	−0.0040 (10)
N2	0.0497 (15)	0.0443 (14)	0.0687 (17)	0.0010 (12)	0.0273 (13)	0.0050 (12)
C1	0.0406 (15)	0.0224 (12)	0.0464 (16)	0.0003 (10)	0.0156 (12)	−0.0020 (10)
C2	0.0416 (15)	0.0307 (13)	0.0447 (15)	−0.0026 (11)	0.0160 (13)	−0.0013 (11)
C3	0.0464 (16)	0.0261 (12)	0.0584 (17)	0.0005 (11)	0.0216 (14)	−0.0032 (12)
C4	0.0461 (16)	0.0333 (15)	0.0586 (18)	0.0050 (12)	0.0212 (14)	−0.0029 (12)
C5	0.0414 (15)	0.0366 (15)	0.0497 (16)	−0.0023 (11)	0.0190 (13)	0.0030 (12)
C6	0.0438 (15)	0.0288 (13)	0.0502 (16)	−0.0012 (11)	0.0137 (13)	−0.0003 (11)
C7	0.0467 (16)	0.0264 (14)	0.0494 (16)	−0.0001 (11)	0.0141 (14)	−0.0006 (12)
C8	0.069 (2)	0.0257 (14)	0.076 (2)	−0.0092 (12)	0.0404 (16)	−0.0070 (13)

### Geometric parameters (Å, °)

**Table:**

O1—C7	1.230 (4)	C1—C6	1.380 (4)
O2—C7	1.319 (3)	C2—C3	1.423 (3)
O3—N2	1.227 (3)	C3—C4	1.357 (4)
O4—N2	1.228 (3)	C4—C5	1.388 (3)
O2—H2	0.8200	C5—C6	1.373 (4)
N1—C8	1.443 (3)	C3—H3	0.9300
N1—C2	1.326 (3)	C4—H4	0.9300
N2—C5	1.437 (3)	C6—H6	0.9300
N1—H1	0.8600	C8—H8A	0.9600
C1—C7	1.466 (3)	C8—H8B	0.9600
C1—C2	1.430 (3)	C8—H8C	0.9600
			
C7—O2—H2	109.00	N2—C5—C6	119.7 (2)
C2—N1—C8	124.3 (2)	C1—C6—C5	121.1 (2)
O3—N2—C5	119.2 (2)	O1—C7—O2	121.4 (2)
O4—N2—C5	118.1 (2)	O1—C7—C1	124.5 (2)
O3—N2—O4	122.7 (2)	O2—C7—C1	114.2 (2)
C2—N1—H1	118.00	C2—C3—H3	119.00
C8—N1—H1	118.00	C4—C3—H3	119.00
C6—C1—C7	119.8 (2)	C3—C4—H4	120.00
C2—C1—C6	119.5 (2)	C5—C4—H4	120.00
C2—C1—C7	120.7 (2)	C1—C6—H6	119.00
N1—C2—C3	119.9 (2)	C5—C6—H6	119.00
N1—C2—C1	122.7 (2)	N1—C8—H8A	109.00
C1—C2—C3	117.4 (2)	N1—C8—H8B	109.00
C2—C3—C4	121.5 (2)	N1—C8—H8C	109.00
C3—C4—C5	119.9 (2)	H8A—C8—H8B	109.00
C4—C5—C6	120.6 (2)	H8A—C8—H8C	110.00
N2—C5—C4	119.6 (2)	H8B—C8—H8C	109.00
			
C8—N1—C2—C1	175.8 (3)	C2—C1—C7—O1	4.1 (4)
C8—N1—C2—C3	−5.1 (4)	C2—C1—C7—O2	−175.9 (2)
O3—N2—C5—C4	176.8 (3)	C6—C1—C7—O1	−178.0 (2)
O3—N2—C5—C6	−2.0 (4)	C6—C1—C7—O2	2.0 (4)
O4—N2—C5—C4	−1.9 (4)	N1—C2—C3—C4	−179.3 (2)
O4—N2—C5—C6	179.4 (3)	C1—C2—C3—C4	−0.1 (4)
C6—C1—C2—N1	179.6 (2)	C2—C3—C4—C5	−0.3 (4)
C6—C1—C2—C3	0.4 (4)	C3—C4—C5—N2	−178.4 (2)
C7—C1—C2—N1	−2.5 (4)	C3—C4—C5—C6	0.4 (4)
C7—C1—C2—C3	178.4 (2)	N2—C5—C6—C1	178.7 (2)
C2—C1—C6—C5	−0.4 (4)	C4—C5—C6—C1	0.0 (4)
C7—C1—C6—C5	−178.3 (2)		

### Hydrogen-bond geometry (Å, °)

**Table:**

*D*—H···*A*	*D*—H	H···*A*	*D*···*A*	*D*—H···*A*
N1—H1···O1	0.86	2.03	2.694 (3)	134
N1—H1···O4^i^	0.86	2.52	3.165 (3)	133
O2—H2···O1^ii^	0.82	1.86	2.679 (3)	177

Symmetry codes: (i) *x*−1, −*y*+1/2, *z*+1/2; (ii) −*x*+1, −*y*+1, −*z*+1.
